# Bi-sinusoidal light stimulation reveals an enhanced response power and reduced phase coherence at the visual cortex in migraine

**DOI:** 10.3389/fneur.2023.1274059

**Published:** 2024-01-11

**Authors:** Thomas C. van den Hoek, Matthijs J. L. Perenboom, Gisela M. Terwindt, Else A. Tolner, Mark van de Ruit

**Affiliations:** ^1^Department of Neurology, Leiden University Medical Center, Leiden, Netherlands; ^2^Department of Human Genetics, Leiden University Medical Center, Leiden, Netherlands; ^3^Department of Biomechanical Engineering, Delft University of Technology, Delft, Netherlands

**Keywords:** visual system, bi-sinusoidal, non-linear, EEG, steady-state response

## Abstract

**Introduction:**

Migraine is associated with enhanced visual sensitivity during and outside attacks. Processing of visual information is a highly non-linear process involving complex interactions across (sub)cortical networks. In this exploratory study, we combined electroencephalography with bi-sinusoidal light stimulation to assess non-linear features of visual processing in participants with migraine.

**Methods:**

Twenty participants with migraine (10 with aura, 10 without aura) and ten non-headache controls were measured (outside attacks). Participants received bi-sinusoidal 13 + 23 Hz red light visual stimulation. Electroencephalography spectral power and multi-spectral phase coherence were compared between groups at the driving stimulation frequencies together with multiples and combinations of these frequencies (harmonic and intermodulation frequencies) caused by non-linearities.

**Results:**

Only at the driving frequency of 13 Hz higher spectral power was found in migraine with aura participants compared with those with migraine without aura and controls. Differences in phase coherence were present for 2nd, 4th, and 5th-order non-linearities in those with migraine (migraine with and without aura) compared with controls. Bi-sinusoidal light stimulation revealed evident non-linearities in the brain’s electroencephalography response up to the 5th order with reduced phase coherence for higher order interactions in interictal participants with migraine.

**Discussion:**

Insight into interictal non-linear visual processing may help understand brain dynamics underlying migraine attack susceptibility. Future research is needed to determine the clinical value of the results.

## 1 Introduction

Migraine is a common paroxysmal brain disorder characterized by recurring attacks of headache that are associated with various autonomic and neurologic symptoms, including hypersensitivity to external sensory inputs such as light (1). An enhanced sensitivity or intolerance to light both during and outside attacks is more prevalent for migraine participants with versus without aura (2), which is in line with the often visual nature of the aura phase. Imaging studies have confirmed the hyperresponsiveness of the visual cortex to light (3–6). The precise mechanisms of a migraine attack and its associated symptoms remain unclear but have been suggested to involve a dynamic change in network excitability throughout the brain, involving cortical and subcortical areas [reviewed in Vecchia and Pietrobon (7), Noseda and Burstein (8), Tolner et al. (9), and Hsiao et al. (10)]. Specifically, the enhanced visual sensitivity in migraine is suggested to involve changes in the integration of visual processing and trigeminal pathways at the subcortical and subsequent cortical level, with converging retino-thalamic and trigeminovascular pathways at the level of the thalamus [(11, 12); for review (13)].

With respect to the brain’s response to external stimuli, a discrimination is made between linear and non-linear processes. Linear interactions between neuronal populations concern neural synchronization at the same frequency, i.e., for which the input frequency (also called “driving frequency”), is the same as the output frequency. In contrast, in nonlinear interactions, synchronization occurs between harmonic frequencies (for which the output frequency is a multiple of the input frequency) and/or intermodulation frequencies (for which multiple input frequencies contribute to one output frequency). For the visual system, the cause of harmonic frequencies is suggested to lie in the resonance of neural processing (14), while intermodulation frequencies are caused by functional integration (15) of visual inputs that involves processing at several levels from the retina to subcortical regions (including the thalamus) to the cortex (16). In the context of all subcortical–cortical interactions involved, the combined linear and non-linear nature of the brain’s processing of visual information could well underlie the highly variable results with electroencephalography (EEG) modalities that have been used to assess the responsivity of the visual cortex to visual stimulation (17, 18). With respect to possible migraine-related changes in the brain’s responsivity to external input, since this involves functional integration of neuronal information at several levels of processing, these are expected to be reflected particularly in intermodulation frequencies in response to external stimuli. Consequently, both linear and non-linear characteristics of the brain’s response to visual stimuli (19) are expected to provide relevant information about migraine-related changes in brain responsivity to external stimuli that can help understand changes in cortical and subcortical processing in the context of migraine susceptibility.

Traditionally, the brain’s response to single visual stimuli is captured using EEG by quantifying features of the visual evoked potential (VEP). Also, repetitive stimuli at a fixed or gradually increasing frequency have been used, causing photic driving, which is the steady-state response measured by EEG at the visual cortex (14). While this photic driving EEG response is dominated by the frequency at which the visual stimuli are presented, the EEG also contains responses at multiples of these frequencies, i.e., the higher harmonics (20). Combining multiple frequencies in one visual stimulation signal, by summing sinusoids at carefully chosen frequencies, results in a more complicated, but richer response at several levels of the visual system, compared to the classic pulse train stimuli. When the visual stimulation signal is composed of multiple frequencies, the output of the non-linear visual system will next to harmonics also present intermodulation frequencies, resulting from the interaction across two or more frequency components of the input signal (21). The origin of sum-of-sinusoid visual stimulation can be traced back to a study in cats that used sums of 6 or 8 frequencies (22). The first study to combine two frequencies in a single visual stimulation signal and record EEG responses from the scalp in humans demonstrated the presence of intermodulation response frequencies (23). Thus, sum-of-sinusoid stimulation reveals not only the harmonic frequencies but also the intermodulation frequencies. Assessing these forms of non-linear responses to visual stimulation provides a way to describe the non-linear properties of the visual system (24) and may provide new quantitative measures of alterations in visual information processing in migraine.

In this explorative study, we compared visual responsivity in participants with migraine and non-headache controls, using bi-sinusoidal light stimulation in combination with EEG. In analyzing the response features, we focused on both the magnitude and phase of the linear and non-linear responses in the EEG at the level of the visual cortex. The goal of this exploratory study was to focus on non-linear interactions in the EEG steady-state response after bi-sinusoidal light stimulation and to explore a new way to assess possible changes in visual processing in migraine.

## 2 Materials and methods

### 2.1 Study design and participants

This was a cross-sectional, non-interventional exploratory study. Participants, aged 18–65 years of both genders (see Table 1), were recruited from the Leiden Headache Center. Both healthy controls and patients with episodic migraine [migraine with aura (MA) and migraine without aura (MO)] fulfilling the criteria of the International Classification for Headache Disorders, 3rd edition (ICHD-3) criteria were included after written informed consent was provided (25). The general exclusion criteria were: (i) psychiatric or neurological disorder other than migraine; (ii) use of chronic medication (other than oral contraceptives), including migraine prophylactics, in the 4 weeks preceding the measurements; (iii) a history of malignancy. Controls were not allowed to suffer from any form of primary or secondary headache other than an occasional simple headache (e.g., low frequent tension-type headache). Patients with migraine had to experience active migraine, which was defined as at least one migraine attack per month in the last 6 months. All EEG recordings took place outside attacks, in an interictal period. Participants were considered to be in the interictal period when they had not experienced a migraine attack within the 72 h prior until 48 h after the recording took place. All participants were screened by telephone for eligibility and were contacted by telephone interview ≥3 days after the experiment to verify interictal status at the time of measurement. The Medical Ethics Committee of the Leiden University Medical Center approved this study under the local protocol number P14.012. All study activities were performed following the Declaration of Helsinki.

**Table 1:** Baseline characteristics of participants: ten non-headache controls, migraine without aura and migraine with aura participants were included in the study.

	Controls (*n* = 10)	Migraine without aura (*n* = 10)	Migraine with aura (*n* = 10)
Female, *n* (%)	7 (70%)	7 (70%)	7 (70%)
Age, mean ± SD	42.2 ± 13.2	42.8 ± 9.2	40.7 ± 9.2
Age at onset of migraine, mean ± SD	–	17.9 ± 5.4	20.3 ± 10.6
Migraine attacks/ month, mean ± SD	–	1.5 ± 0.7	1.4 ± 1.2
Migraine days per month, mean ± SD	–	2.9 ± 2.1	2.3 ± 2.4
Use of triptans, *n* (%)	–	5 (50%)	6 (60%)
Migraine attacks with aura, % of total number of attacks	–	–	74.5

Migraine patients had 1–2 attacks, accounting for 2–3 migraine days, per month. The migraine with aura participants experienced an aura in on average 74.5% of their attacks.

### 2.2 Procedures

All participants visited the Leiden Headache Center for EEG recording while receiving bi-sinusoidal light stimulation on a single occasion. Recordings took place between 9 AM and 5 PM. During the recordings, participants lay on a bed with their eyes closed in a darkened and sound-deprived room. For the EEG recordings, a total of seven Ag-AgCl electrodes were used located at Fz, Cz, C3, C4, Oz, O1 and O2 of the 10–20 system. EEG was online referenced to the C3 and C4 electrodes (EEG-1200; Nihon Kohden, Tokyo, Japan). Data were sampled at 1000 Hz and band-pass filtered online between 0.08 and 300 Hz.

Continuously-modulated light stimulation was provided to the patient using binocular goggles with red (654 nm) LED lights (Synergy Plinth, Medelec International) while the participants had their eyes closed. Red light was found to effectively activate cone-driven retinal pathways and result in prominent cortical and thalamic neuronal responses (26). The intensity and frequency of light stimulation were determined by an analog output current generated from a National Instruments Data Acquisition Device (NI-9265 C-series 16-bit current output module) and controlled through a custom-written MATLAB script. In this exploratory study, the maximal possible LED light intensity was used (438 cd/m^2^) (20).

**Figure 1:**
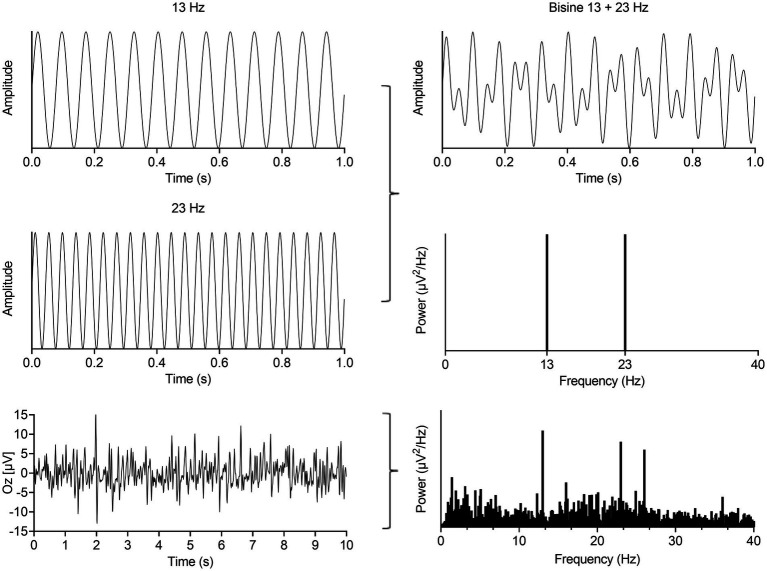
Summation of a 13 and 23 Hz sine (left, up and middle) results in a bi-sinusoidal signal which can be represented in time- (top, right) and frequency-domain (right, middle). This signal was used as the light stimulation signal. A representative EEG recording from the occipital cortex in time- (left, bottom) and frequency-domain (right, bottom) during bi-sinusoidal light stimulation shows clear activity at the driving (13 and 23 Hz) and a harmonic frequency (2 × 13 Hz = 26 Hz).

For this study, a bi-sinusoidal continuous light stimulation was designed consisting of frequencies 13 and 23 Hz (Figure 1). Effectively, two sine waves with a frequency of 13 and 23 Hz, with random phase, were summed and scaled to modulate a light intensity with a modulation depth of 60%. The frequencies of 13 and 23 Hz, further referred to as driving frequencies, were chosen to avoid overlap of interactions between both frequencies and allow assessment of non-linear EEG responses up to the 10th order (Table 2). The sinusoidal light stimulations were presented in 10 blocks of 32 s, with 5 to 10 s rest in between blocks. A recording session took ~30 min, including the intake (~10 min), EEG preparation (~10–15 min) and light stimulation protocol (~7 min).

**Table 2:** Harmonic and intermodulation frequencies up to and including the 5th order for an input signal consisting of a 13 and 23 Hz sine wave.

Order	Type of non-linearity	Frequency combinations (r, l = 1,2 and r ≠ l)	Output frequency *f*_Σ_ (Hz) (*f*_Σ_ ≥ 0)
2	Harmonics	2 *f_r_*	26, 46
	Intermodulation	*f_r_* ± *f_l_* > 0	10, 36
3	Harmonics	3 *f_r_*	39, 69
	Intermodulation	2 *f_r_* ± *f_l_* > 0	3, 33, 49, 59
4	Harmonics	4 *f_r_*	52, 92
	Intermodulation	3 *f_r_* ± *f_l_* > 0	16, 20, 56, 62, 72, 82
	Intermodulation	2 *f_r_* ± 2 *f_l_* > 0	20, 72
5	Harmonics	5 *f_r_*	65, 115
	Intermodulation	3 *f_r_* ± 2 *f_l_* > 0	43, 75, 95
	Intermodulation	4 *f_r_* ± *f_l_* > 0	7, 29, 43, 75, 79, 85, 95, 105

### 2.3 Data pre-processing and analysis

All data processing and analyses were performed in MATLAB (Version R2020b, The Mathworks, Natick, MA, United States). First, EEG data were band-pass filtered between 1 and 250 Hz (3rd order Butterworth filter) and bad channels were removed based on a channel’s kurtosis and variance (removed when z-score < −3 or > 3). Second, the ten 32 s blocks during which participants received light stimulation were extracted from the EEG data and segmented into 1 s time-locked epochs. Visual inspection was used to confirm the absence of any muscle- or movement-related artifacts. This resulted in the use of 320 epochs that were all used for the subsequent analysis.

Two parameters were calculated for analyzing changes in the visually-evoked steady-state response at electrode Oz. Only Oz was used as we previously demonstrated that at this electrode we find the strongest response to the driving frequencies, and best signal-to-noise ratio, when using flash stimulation (20). First, we determined the spectral power of the steady-state response using the Fourier Transform over the 1 s epochs multiplied with a Hanning-window. Second, the consistency of non-linear cross-frequency phase difference across epochs between the light stimulation signal and steady-state response at Oz was calculated using the multi-spectral phase coherence (MSPC) method (24). MSPC is a general method to calculate phase coupling of various order non-linearities between two signals. In short, with d-th order non-linearity and two-time series x(t) and y(t) with X(f) and Y(f) representing their Fourier transforms, MSPC of K number of epochs is defined by:


$$\begin{array}{l} \Psi_{XY}{\left(f_{1},\;f_{2},\;\ldots ,\;f_{R};a_{1},\;a_{2},\;\;\ldots ,\;\;a_{R}\right)}_{d}\\ =\frac{1}{K}\overset{K}{\underset{k=1}{\sum }}\exp\left(j\left(\overset{R}{\underset{r=1}{\sum }}a_{R}\phi_{X_{k}}\left(f_{R}\right)-\phi_{Y_{k}}\left(f_{\Sigma }\right)\right)\right) \end{array}$$

where $f_{1},f_{2},\ldots ,f_{R}$ are frequencies of *X(f)*; $a_{1},a_{2},\ldots ,a_{R}$ are the integer weights of these frequencies, and $\phi_{X_{k}}\left(f_{R}\right)$ is the phase of $X\left(f_{r}\right)$ at *k-*th epoch. Similarly, $\phi_{Y_{k}}\left(f_{\Sigma }\right)\;$ is the phase of an output frequency in Y(f) that is a combination of input frequencies with the order of non-linearity $\;d=\overset{R}{\underset{r=1}{\sum }}\left|a_{r}\right|>2$ (*d* = 1 indicating linear interaction). Hereby, MSPC is used to quantify the *d-*th order harmonic ( $d\cdot f_{r}$ ) and intermodulation coupling ( $\overset{R}{\underset{r=1}{\sum }}a_{r}f_{r},R\geq 2,\overset{R}{\underset{r=1}{\sum }}\left|a_{r}\right|=d$ ) between x(t) and y(t) . The magnitude of MSPC is defined as the multi-spectral phase coherence, denoted as $\psi =\left|\Psi \right|$ . This multi-spectral phase coherence reflects the strength of non-linear phase coupling and varies between 0 and 1. Here, 0 indicates a random non-linear phase relationship, and 1 indicates a perfectly consistent non-linear phase relationship across epochs (24). In the present work x(t) is the light pattern signal, whereas y(t) is the recorded steady-state response from Oz.

### 2.4 Statistical analysis

No sample size calculation was performed for this study due to the explorative nature of the experiment and the lack of previous studies using bi-sinusoidal light stimulation in migraine patients. The descriptive statistics of the study participants, including migraine characteristics, are presented using means and standard deviations. All data were confirmed to be normally distrubed using normality tests. Therefore, for the EEG parameters, spectral power was compared across groups (MA, MO and controls) using a one-way ANOVA.

Analysis for phase coherence was performed up to the tenth order non-linear interactions for spectral power, and for MSPC up to the highest order at which MSPC was still significant. The significance level for MSPC was determined at $\sqrt{3/K},$ in which *K* is the number of epochs (24), under the null-hypothesis that there are no interactions between frequencies with a 95% confidence interval. Results from MSPC were compared across groups using one-way ANOVA.

All results for the ANOVA F-tests were considered significant at the 5% level (*p* = 0.05). Any significant differences across subgroups (e.g., MA vs. MO, MA vs. controls) were further explored using closed testing procedures as *post-hoc* testing procedure (27), in this case using unpaired *t*-tests across subgroups. The chosen closed testing procedure for *post-hoc* testing in ANOVA is especially applicable for 3 groups and is more powerful than Bonferroni while controlling the same stringent error rate (27). Statistical analyses were conducted in SPSS version 25.0 for Windows (IBM, Armonk, NY, United States).

### 2.5 Data availability

The data that support the findings of this study are available from the corresponding author, upon reasonable request.

## 3 Results

### 3.1 Participants

For this exploratory study, a total of 30 participants were included, matched for sex and age: 10 non-headache controls and 20 patients with migraine (10 MA; 10 MO; Table 1). All participants completed the study.

### 3.2 Enhanced visual evoked EEG power for MA participants at the 13 Hz driving frequency

**Figure 2:**
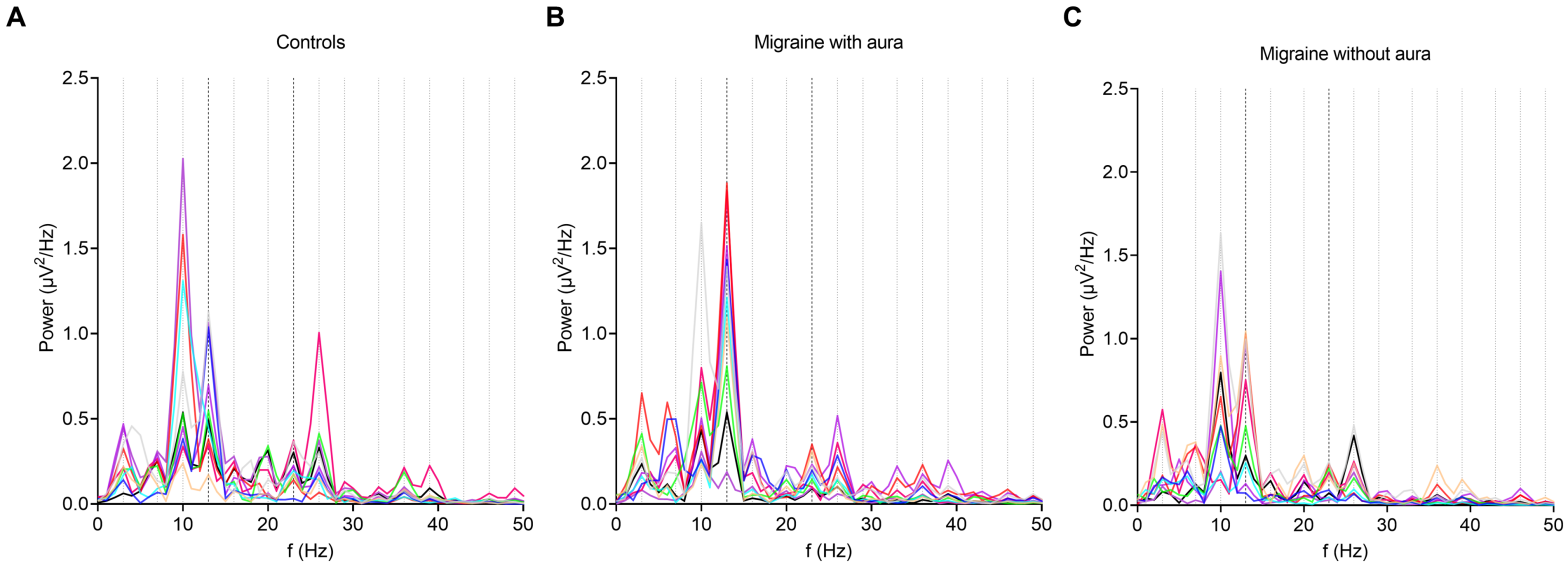
Overall response power changes from baseline after bi-sinusoidal light stimulation. In all graphs, the presence of an EEG response to bi-sinusoidal stimulation is evident at driving (13 and 23 Hz) and harmonic frequencies. A decrease in overall power can be observed at higher frequencies (shown to a maximum of 50 Hz). Stimulation frequencies (13 and 23 Hz) and harmonic frequencies up until and including the 5th order are shown as black and grey dotted lines on the *x*-axis at corresponding frequencies, respectively. Each line represents a single participant. **(A)** controls; **(B)** participants with migraine with aura; **(C)** participants with migraine without aura.

**Figure 3:**
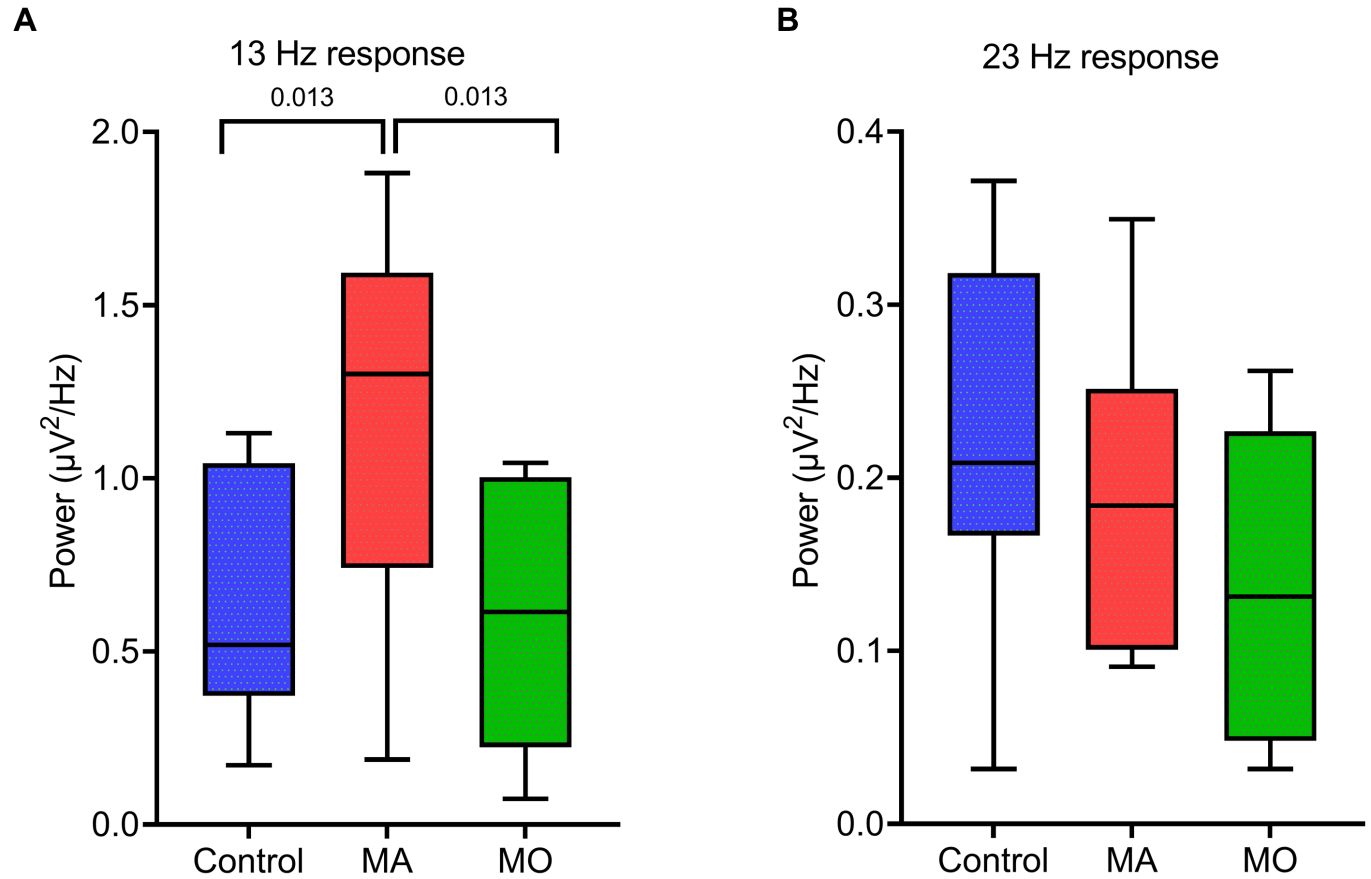
EEG spectral power for the response at driving frequencies (13 and 23 Hz) following bi-sinusoidal light stimulation for all groups. **(A)** MA vs. MO at 13 Hz response. **(B)** Control vs. MA vs. MO at 23 Hz response. Significant *p*-values after unpaired *t*-tests (closed testing procedure) are depicted.

During bi-sinusoidal light stimulation of summed 13 and 23 Hz sine waves (Figure 1), power spectral analysis of the steady-state response at the visual cortex (Oz) revealed a spectral response at the driving frequencies of 13 and 23 Hz for MA, MO and controls (Figure 2). Comparing MA, MO and controls, using a one-way ANOVA with a *post-hoc* closed testing procedure (Figures 3A,B), revealed a difference in spectral power between MA, MO and controls for 13 Hz (*F*(2,27) = 5.8, *p* = 0.008). No differences in steady-state response power across groups were found at 23 Hz nor the harmonics and intermodulation frequencies of the driving frequencies. An independent *t*-test used in a closed testing procedure revealed higher steady-state response power for 13 Hz in MA compared to MO participants (mean difference, 0.59, 95%CI, 0.14–1.03; *p* = 0.013) and between MA and controls (mean difference, 0.56, 95%CI, 0.13–0.98; *p* = 0.013). No difference was found between MO and controls (mean difference − 0.03, 95%CI, −0.37–0.32; *p* = 0.86).

### 3.3 Lower MSPC at non-linear response frequencies in migraine compared to controls

**Figure 4:**
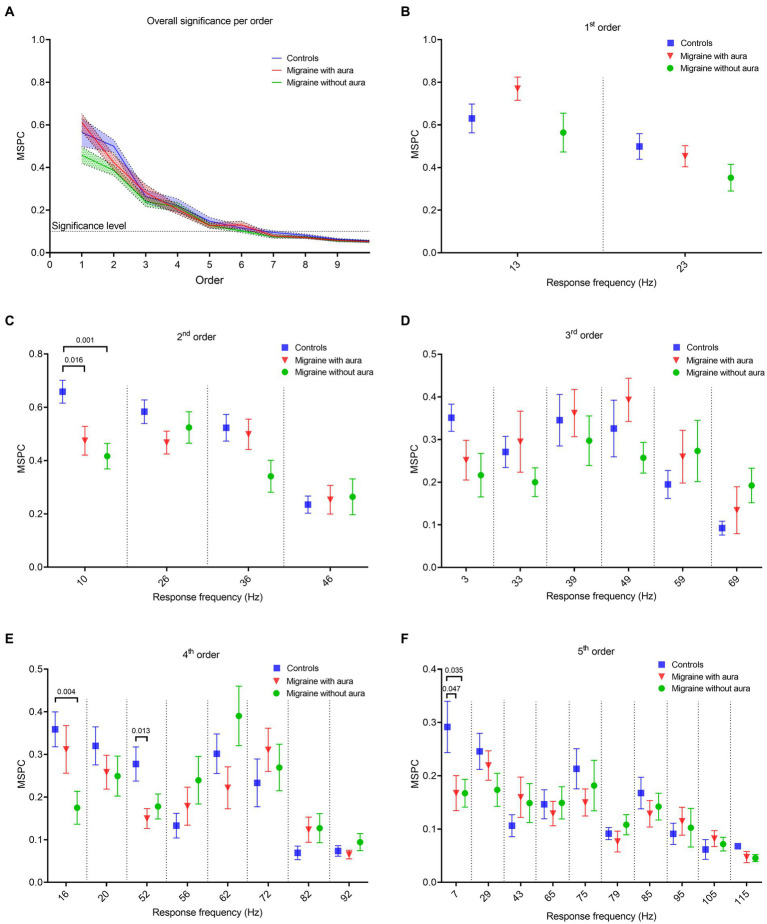
Phase coherence features between the bi-sinusoidal light stimulation signal and the EEG at Oz for all groups, for driving, harmonic and intermodulation frequencies. **(A)** Consistency of the (non-)linear cross-frequency phase difference across epochs between the bi-sinusoidal light stimulation signal and EEG response at electrode Oz calculated by the multi-spectral phase coherence (MSPC) method. Significant non-linear interactions at Oz are found up to the 5th order for all groups (blue: controls, red: MA, green: MO). **(B–F)** MSPC at driving, harmonic and intermodulation frequencies up to and including the 5th order. Harmonic and intermodulation frequencies differ per order (see Table 2 for an overview of all included frequencies up till and including the 5th order).

The consistency of the non-linear cross-frequency phase difference across epochs between the bi-sinusoidal light stimulation signal and EEG steady-state response at Oz was calculated using the multi-spectral phase coherence (MSPC) method (24). A significant MSPC (MSPC >0.097) was found for MA, MO and controls up to the fifth order (Figure 4A).

Phase coherence, calculated using the MSPC, for the response at the first order driving frequencies (13 and 23 Hz) was not different between MA, MO and controls (Figure 4B). However, differences (one-way ANOVA with a *post-hoc* closed testing procedure) in non-linear interactions were found at harmonic and intermodulation frequencies up to, and including, the 5th order (Table 2 and Figures 4C–F). Specifically, a lower phase coherence was found for 2nd order interactions at 10 Hz, for MA as well as MO compared to controls (MA: mean difference, −0.18, 95%CI, −0.33 – −0.04; *p* = 0.016; MO: mean difference, −0.24, 95%CI, −0.37 – −0.11; *p* = 0.001). The 4th-order interactions at 16 Hz also demonstrated a significantly lower phase coherence for MO to controls (mean difference, −0.18, 95%CI, −0.30 – −0.07; *p* = 0.004). Furthermore, a lower phase coherence was observed at 52 Hz for MA compared to controls (mean difference, −0.13 95%CI, −0.23 – −0.03 *p* = 0.013). Finally, MSPC was lower at the 5th order interactions at 7 Hz for MA compared to controls (mean difference, −0.12, 95%CI, −0.25 – −0.001; *p* = 0.047) and for MO compared to controls (mean difference, −0.12, 95%CI, −0.24—0.01; *p* = 0.04). No differences in phase coherence were found for 3rd order interactions. Generally, distinct reductions in phase coherence were observed for 2nd, 4th, and 5th order non-linear interactions for MA and MO groups compared to controls.

## 4 Discussion

In this exploratory study, we exploit a new method to compare visual responsivity, quantified using the steady-state response to bi-sinusoidal light stimulation, in migraine participants with (MA) and without aura (MO) and non-headache controls. The use of a continuous light stimulation signal composed of the sum of two driving frequencies, 13 and 23 Hz, enabled analysis of both magnitude and phase of the linear as well as nonlinear responses – i.e. at harmonic and intermodulation frequencies – in the steady-state response at the level of the visual cortex. A higher spectral power was found for the 13 Hz driving frequency in MA compared to MO and controls together with a lower MSPC at non-linear interaction frequencies in both migraine groups compared with controls. Our findings show that the use of a carefully designed visual stimulation signal consisting of a sum of two sine waves allows the quantification of differences in both linear and non-linear response features of the migraine brain.

### 4.1 Sum-of-sine visual stimulation allows for studying linear and non-linear interactions within the migraine brain

This study is the first to explore non-linear response characteristics of the migraine brain to visual stimulation not only at harmonic but also at intermodulation frequencies. The use of a continuous (sum-of-)sine wave modulated light source allows us to obtain a more complete picture of the visual system’s network dynamics in the context of migraine. Traditional SSVEP studies only presented visual stimuli at a single fixed frequency, thereby only revealing changes in the stimulation driving frequency and its harmonics. Moreover, the use of repetitive flash or checkerboard on/off paradigms results in a square wave input signal, which effectively is a broadband sum-of-sine signal composed of many sinusoids of many frequencies, that does not enable separating linear and non-linear dynamics. Our visual stimulation signal was composed of only two carefully selected frequencies, the alpha (13 Hz) and beta (23 Hz) band, that have been used in various visual stimulation studies in migraine based on their photic driving capabilities (28–33). Since these frequencies do not cause any overlap in frequencies caused by harmonic responses and intermodulation, we speculate that the bi-sinusoidal stimulation input signal used in this study will allow to separate mechanisms believed to contribute to linear and non-linear processing steps of visual information within the neuronal pathways from the retina to the thalamus to the cortex, such as (visual) neural resonance (14) and functional integration (15).

### 4.2 Enhanced EEG response power at driving frequencies for MA

The spectral power of the steady-state response recorded over the visual cortex (Oz electrode) revealed distinct response peaks at both driving (13 and 23 Hz), harmonic (integer multiples of driving frequencies, e.g., 26 Hz) and intermodulation (interactions between driving frequencies, e.g., 23 Hz −13 Hz = 10 Hz) frequencies. These findings confirm the presence of both linear and non-linear response characteristics of the visual system (14, 19). Yet, in our study spectral power was only found to be enhanced for the 13 Hz driving frequency in MA. Our findings align with earlier reports that used repetitive light flashes or continuous modulated light to assess altered visual responsivity using EEG in migraine. Enhanced spectral power at the driving frequency in the cortical steady-state response to repetitive light stimulation for participants with migraine has been reported earlier (29, 32, 34, 35), or specifically for MO (31, 36, 37). Enhanced excitability at cortical and/or subcortical levels – including reticulo-thalamic hyperexcitability – were suggested as possible underlying mechanisms. In contrast, other studies did not find a difference in driving response for migraine (20), or observed a reduced driving response for MO (28). In which way the enhanced response power for 13 Hz in MA may reflect enhanced cortical inhibition, as indicated for the EEG alpha rhythm (38), possibly as a consequence of generally enhanced neuronal excitability in MA, requires further studies, e.g., using paired-pulse paradigms.

We did not find any differences in occipital cortex spectral power for harmonic or intermodulation frequencies in participants with MO and MA, in line with a previous study that did not find differences at the 2nd order harmonics in response to 12–27 Hz flash stimulation (32). Differences have been reported by others, with one study reporting a reduced spectral power for MO but not MA for the 2nd order harmonics (31), while another reported an enhanced power of the 2nd order harmonic response for MA and MO as well as an enhanced response power at higher (4th order) harmonic frequencies for MA (39). These seemingly conflicting observations, for both linear and non-linear responses to light stimulation, can be explained by the heterogeneity of methodologies including possible effects of eye closure or opening on alpha-beta frequency responses.

### 4.3 Lower MSPC for non-linear response frequencies in migraine

We observed a lower MSPC in migraine participants for non-linear interactions up to the 5th order. The phase coherence was quantified between the visual input signal, i.e., the sum-of-sine signal composed of 13 and 23 Hz, and the steady-state response recorded at the occipital cortex. Our finding highlights reduced synchrony between the visual input and the brain’s response to this input in migraine, specifically at harmonic and intermodulation frequencies. At the network level, our findings suggest a specifically altered contribution of higher-order visual processing to the overall steady-state response. Besides altered cortico-cortical interactions, this may involve altered dynamics among subcortical thalamic and cortical regions in migraineurs as suggested based on clinical (12, 40) and preclinical findings (13, 41).

Whereas our measure of coherence relates the visual cortex EEG to the phase of the external stimuli, more commonly changes in resting-state cortico-cortical phase coherence (or phase synchrony) in migraine have been studied. Reduced phase coherence between the left and right occipital cortex was reported for the theta, gamma, alpha, and beta band in MO during the interictal migraine phase (42). Similarly, for MA lower interhemispheric coherence between O1 and O2 for the alpha band was reported, while various intra-hemispheric coherence features were enhanced (43). Reduced phase coherence in MA may reflect altered synchronization within visual system networks. In line with this idea, others reported decreased interhemispheric occipital phase synchronization for EEG recorded during visual stimulation in the beta band in MA, while in MO phase synchronization was increased for the alpha band (30, 44).

### 4.4 Future directions

Compared to classical flashing light stimulation, the use of bi-sinusoid stimulation provides an advantage by allowing the identification of nonlinear visual response differences for both harmonic and intermodulation frequencies. Shedding further light on characteristics of linear and nonlinear responses of the brain to visual stimuli in the context of migraine may in the future also help to understand the large heterogeneity in findings of migraine EEG studies to date. This will require an extended set of data from larger groups of patients that would allow correlating linear and nonlinear visual response features not only to migraine subtypes (MA and MO) but also to other clinical disease variables. In addition, the selection of different sets of stimulation frequencies in addition to the currently used two frequencies will likely be important to uncover specific dynamics of the migraine brain, given the complex brain-wide network responses to naturally occurring visual inputs (45, 46). Physiologically inspired computational EEG models that account for subcortical–cortical as well as cortico-cortical connectivity (47, 48) could help to interpret (linear and nonlinear) findings and optimize future EEG study designs to uncover migraine-specific brain activity dynamics. Finally, employing longitudinal measurements across the migraine cycle would be a valuable addition to provide insight into changes in linear and nonlinear visual responsivity in the context of migraine attack occurrence.

## Data availability statement

The dataset presented in this article is available from the corresponding authors, upon reasonable request. Requests to access the datasets should be directed to ET, e.a.tolner@lumc.nl | MR, M.L.vandeRuit-1@tudelft.nl.

## Ethics statement

The study involved humans and was approved by Medical Ethics Committee of the Leiden University Medical Center. The study was conducted in accordance with the local legislation and institutional requirements. The participants provided their written informed consent to participate in this study.

## Author contributions

TH: Data curation, Formal analysis, Investigation, Project administration, Visualization, Writing – original draft, Writing – review & editing. MP: Conceptualization, Investigation, Methodology, Software, Writing – review & editing. GT: Supervision, Writing – review & editing. ET: Supervision, Writing – review & editing, Conceptualization. MR: Formal analysis, Supervision, Writing – review & editing, Investigation, Software.
